# Investigate the effect of oxygen-driven salbutamol combined with methylprednisolone on bronchial asthma from the perspective of serum molecular markers

**DOI:** 10.5937/jomb0-59800

**Published:** 2026-01-28

**Authors:** Lu Qi, Xiao Zhang, Yuanyuan Ju, Xin Yang

**Affiliations:** 1 Children's Medical Center, Second Hospital of Shandong University, Jinan, China

**Keywords:** salbutamol, methylprednisolone, bronchial asthma, inflammatory factors, stress response, immune globulin, salbutamol, metilprednizolon, bronhijalna astma, inflamatorni faktori, stresni odgovor, imunoglobulin

## Abstract

**Background:**

This study explores the application of oxygen-driven salbutamol (SA) combined with methylprednisolone (MP) in bronchial asthma (BA) from the perspective of objective clinical markers, including serum inflammatory response, oxidative stress response, immunoglobulin, and pulmonary ventilation function.

**Methods:**

A retrospective analysis was performed on 207 pediatric BA patients admitted to our hospital between January 2022 and January 2025. Of them, 114 children received MP combined with SA nebulization (control group), and 93 children received MP combined with oxygen-driven SA (observation group). The pulmonary ventilation function, inflammatory mediators (HIF-1a, IL-4, IL-6, IL-8 and TNF-a), stress response indicators (SOD, NO, ET-1 and MDA) and immunoglobulin (IgE, IgA, IgM and IgG) were detected and compared before and after treatment.

**Results:**

Post-treatment, the observation group demonstrated superior pulmonary ventilation function and significantly lower levels of inflammatory mediators (HIF-1a, IL-4, IL-6, IL-8, and TNF-a) compared to the control group (P &lt; 0.05). Regarding stress response, the SOD level in the observation group was higher after treatment, while the levels of NO, ET-1, and MDA were lower (P &lt; 0.05). In addition, IgE was lower and IgA was higher in the observation group than in the control group after treatment (P &lt; 0.05).

**Conclusions:**

The combination of oxygen-driven SA and MP is more effective in mitigating inflammatory, stress responses and optimizing immune function in pediatric BA patients.

## Abstract (sr)

**Uvod:**

Ova studija istražuje primenu salbutamola (SA) aktiviranog kiseonikom u kombinaciji sa metilprednizolonom (MP) kod bronhijalne astme (BA) iz perspektive objektivnih kliničkih markera, uključujud serumski inflamatorni odgovor, odgovor na oksidativni stres, imunoglobulin i funkciju plucne ventilacije.

**Metode:**

Retrospektivna analiza je sprovedena na 207 pedijatrijskih pacijenata sa BA primljenih u našu bolnicu između januara 2022. i januara 2025. godine. Od njih, 114 dece je primalo MP u kombinaciji sa nebulizacijom sa sarkom (kontrolna grupa), a 93 dece je primalo MP u kombinaciji sa sarkom pokretanom kiseonikom (posmatračna grupa). Funkcija plucne ventilacije, inflamatorni medijatori (HIF-1a, IL-4, IL-6, IL-8 i TNF-a), indikatori odgovora na stres (SOD, NO, ET-1 i MDA) i imunoglobulini (IgE, IgA, IgM i IgG) su detektovani i upoređeni pre i posle lečenja.

**Rezultati:**

Nakon tretmana, posmatrana grupa je pokazala superiornu funkciju plucne ventilacije i značajno niže nivoe inflamatornih medijatora (HIF-1a, IL-4, IL-6, IL-8 i TNF-a) u poređenju sa kontrolnom grupom (P &lt; 0,05). Sto se tiče odgovora na stres, nivo SOD u posmatranoj grupi je bio viši nakon tretmana, dok su nivoi NO, ET-1 i MDA bili niži (P &lt; 0,05). Pored toga, IgE je bio niži, a IgA viši u posmatranoj grupi nego u kontrolnoj grupi nakon tretmana (P &lt; 0,05).

**Zaključak:**

Kombinacija kiseoničnog sarkoma i mikroplazmatskog antacida je efikasnija u ublažavanju inflamatornih i stresnih reakcija i optimizaciji imunološke funkcije kod pedijatrijskih pacijenata sa BA.

## Introduction

Bronchial asthma (BA), a chronic inflammatory airway disorder, arises from the interplay of multiple inflammatory mediators and cytokines. Marked by persistent airway inflammation and heightened responsiveness, BA is notorious for its recurrent episodes [1]. A cross-sectional survey on BA by Shamshad T et al. [2] showed that the prevalence of BA was as high as 26.9% in children aged 6-16 years, and its prevalence showed an increasing trend from year to year. Clinically, the cornerstone of BA management lies in glucocorticoids, with methylprednisolone (MP) being a prominent choice [3]. Extensive research has validated MP's potent anti-allergic, anti-inflammatory, and immunosuppressive properties. Moreover, it can achieve peak plasma levels within a mere 30 minutes, rendering it pivotal in alleviating acute exacerbations of BA [4]. Nonetheless, MP alone falls short in curbing BA's progression. A comprehensive approach necessitates not only MP but also strategies to alleviate bronchospasm and thwart airway remodeling [5]. Salbutamol (SA) is a β2-adrenergic receptor agonist renowned for its antitussive prowess. When harnessed through oxygen-driven nebulization-a technique leveraging high-velocity oxygen streams to create negative pressure for drug atomization-SA particles, accompanied by oxygen, penetrate deep into the airways. This dual action not only amplifies therapeutic outcomes but also ensures oxygenation [6]. While the literature abounds with accounts of MP and SA coadministration for BA, but we found that these studies mainly focused on the clinical effect of MP combined with SA in the treatment of BA [7] [8], and few explored the role of MP combined with SA from the perspective of objective clinical indicators.

Delving deeper into BA's pathogenesis, contemporary insights reveal an intricate association between inflammatory mediators, notably interleukins (ILs), and a plethora of inflammatory cells [9]. For instance, the rhythmic oscillation of IL-6 induced by brain and muscle arnt-like protein 1 (BMAL1)/forkhead box A2 (FOXA2) has been implicated in nocturnal BA exacerbations [10]. Similarly, IL-4 variants may exacerbate BA severity via Treg cell-mediated pathways [11]. These findings underscore the necessity, in contemporary BA treatment research, of not only closely monitoring patients' clinical manifestations and pulmonary function improvements but also placing significant emphasis on alterations in inflammatory mediators like ILs. However, few studies have elucidated the mechanistic effects of this combination therapy on systemic inflammatory and oxidative stress biomarkers in pediatric populations.

In this context, this study explores the application of oxygen-driven SA combined with MP in BA from the perspective of objective clinical markers, including serum inflammatory response, oxidative stress response, immunoglobulin, and pulmonary ventilation function. The results of this study will provide more accurate guidance for the clinical application of oxygen-driven SA combined with MP in the future.

## Materials and methods

### Study design

This study was designed as a retrospective analysis, focusing on pediatric BA patients admitted to Second Hospital of Shandong University between January 2022 and January 2025. The sample size was determined using G-Power software (Two-tailed test, effect 0.3, α 0.5, power 0.8), followed by a rigorous screening process based on predefined inclusion and exclusion criteria, as outlined below:

Inclusion criteria: (1) A confirmed diagnosis of BA [(1) recurrent episodes of wheezing, coughing, shortness of breath, or chest tightness triggered by exposure to cold air, physical stimuli, or chemical irritants, respiratory infections, exercise, or hyperventilation; (2) the presence of scattered or diffuse wheezing sounds, primarily during expiration; (3) At least one of the following objective criteria: a positive bronchial provocation or exercise challenge test; or Positive bronchial provocation test; or a daily variability in peak expiratory flow of ≥ 13%] [12]. (2) Treatment with MP combined with SA during hospitalization at our institution. (3) Age under 14 years. (4) Availability of complete medical records.

Exclusion criteria: (1) A history of drug allergies. (2) Concurrent severe organic diseases or baseline pulmonary dysfunction not related to the current asthma attack. (3) Poor adherence to treatment protocols, rendering completion of the prescribed therapy unfeasible.

### Ethical considerations

Approval for this study was granted by the Ethics Committee of Second Hospital of Shandong University. As it was a retrospective analysis, informed consent was waived for this study.

### Study participants

Following the screening process, 207 pediatric BA patients were enrolled. Of these, 114 were assigned to the control group, receiving MP combined with SA nebulization, while the remaining 93 were allocated to the observation group, receiving MP combined with oxygen-driven SA therapy.

### Treatment protocol

All children were treated with intravenous MP (Tianjin Jin Yao Pharmaceutical Co., Ltd, H20123319), 2 mg/kg, diluted with 50 mL of 5% dextrose solution, 2-3 times/d for 5 consecutive days after admission, infused over 30 minutes.

Control Group: Patients in this group underwent SA (GlaxoSmithKline Australia Pty Ltd, HJ20160660) nebulization therapy. The dosage was adjusted based on body weight: 2.5 mg per session for children weighing 20 kg and 5 mg per session for those>20 kg. Nebulization was carried out using an SN-type ultrasonic nebulizer (Tianjin Shengning Biotech Co., Ltd., SN-007-C), with each session lasting 15-20 min, 2 times/d, for a total of 5 days.

Observation Group: In addition to the above treatment, this group received oxygen-driven SA therapy. The treatment involved connecting oxygen equipment (Hebei Jinweikang Medical Equipment Co., Ltd, 20182080242) to an oxygen flow meter and a disposable jet nebulizer, with the oxygen flow rate maintained at 5-8 L/min. Each inhalation session lasted 15-20 minutes and was conducted twice daily for 5 days.

### Pulmonary function testing

Pulmonary function testing was performed using a MasterScreen spirometer (Care Fusion Germany 234GmbH, SFDA (I) 20122213885) according to ATS/ERS guidelines. Patients were instructed to perform three forced expiratory maneuvers, and the highest value of forced expiratory volume in 1 second (FEV1), forced vital capacity (FVC), and peak expiratory flow (PEF) was recorded.

### Laboratory testing

Arterial and venous blood samples were collected from participants before and after treatment. Arterial blood was analyzed for blood gas parameters (Radiometer, ABL90), including oxygen saturation of blood (SaO_2_), partial pressure of oxygen (PaO_2_), and arterial partial pressure of carbon dioxide (PaCO_2_).

Venous blood samples were subjected to enzyme-linked immunosorbent assay (ELISA) to quantify levels of hypoxia-inducible factor 1α (HIF-1α), IL-4, IL-6, IL-8, Tumor necrosis factor-α (TNF-α), nitric oxide (NO), endothelin 1 (ET-1), and malondi-aldehyde (MDA). All ELISA kits were sourced from Beijing TransGen Biotech Co., Ltd. Procedure: 50 μL of standards (seven gradients, 0.1-1000 pg/mL) and samples (50 μL) were added to each well, and the edge wells were plate sealed with washing solution. Incubation was performed at 37°C for 90 min (shortened to 60 min due to poor stability of HIF-1α) to allow adequate antigen/antibody binding. Unbound material was removed by washing 5 times (30 s each) with a plate washer. 100 μL of biotin-labeled antibody was added to each well and incubated at 37°C for 60 min. After washing, 100 μL horseradish peroxidase-streptavidin was added to each well and incubated at 37°C for 30 min. 100 μL of 3, 3', 5, 5' tetramethyl-benzidine (TMB) substrate was added to each well and incubated in the dark for 15 min (the color development time for HIF-1α should be shortened to 10 min). 50 μL of termination solution (sulfuric acid) was added to each well, and optical density (OD) at 450 nm was immediately read using a microplate reader. The standard curve (four-parameter Logistic fitting) was drawn with the OD value of the standard as the abscordinate and the concentration as the ordinate. The concentration (pg/mL) was checked by the OD value of the sample. Quality control: high, medium and low concentrations of quality control serum were added to each test (the same batch as the sample), and Levey-Jennings quality control chart was drawn. The quality control values of 20 consecutive tests were required to be within ±2 standard deviation (SD).

Immunoglobulin (Ig) E, M, G and A were detected by immune transmission turbidimetry with Mindray BS-230 instrument. Operation procedure: Calibrate the instrument with the matching calibrator (the concentration covers the detection range, such as IgG 5-16 g/L) and input the concentration value of the calibrator. Quality control: Test the high, medium and low concentration quality control materials (the same batch as the calibrators), and confirm the results are in control (CV 5%). The sample was added to the reaction cup. Anti-human Ig antibody reagent (ratio 1: 20) was added and incubated at 37°C for 5 min. The scattered light intensity at 340 nm was measured (rate method), and the sample concentration (g/L) was automatically calculated from the calibration curve.

### Statistical analysis

Data analysis was conducted using SPSS 26.0. Categorical variables, expressed as frequencies and percentages [n (%)], were compared using the chi-square test. Continuous variables were assessed for normality using the Shapiro-Wilk test. For normally distributed data, presented as mean ± standard deviation (χ^2^±s), comparisons were made using independent samples t-test and paired t-test. A P-value of less than 0.05 was considered statistically significant.

## Results

### Comparability of study participants

A comparative analysis of baseline characteristics, including age, duration of BA, and gender, revealed no statistically significant differences between the two groups (*P* > 0.05). This finding underscores the comparability of the two groups (Table 1).

**Table 1:** Comparison of clinical baseline information. Note: Bronchial asthma (BA).

Projects	Control (n=114)	Observation (n=93)	Statistical	P
Age	6.78±1.46	6.72±1.36	t=0.304	0.761
Male/Female	61 (53.51%)/<br>53 (46.49%)	55 (59.14%)/<br>38 (40.86%)	χ^2^=0.659	0.417
Weight (kg)	23.68±3.17	24.37±3.41	t=1.486	0.139
Duration of BA (months)	17.04±7.52	17.77±7.00	t=0.726	0.469
Family history of disease in BA	26 (22.81%)	28 (30.11%)	χ^2^=0.234	1.416

### Pulmonary function

**Figure 1:**
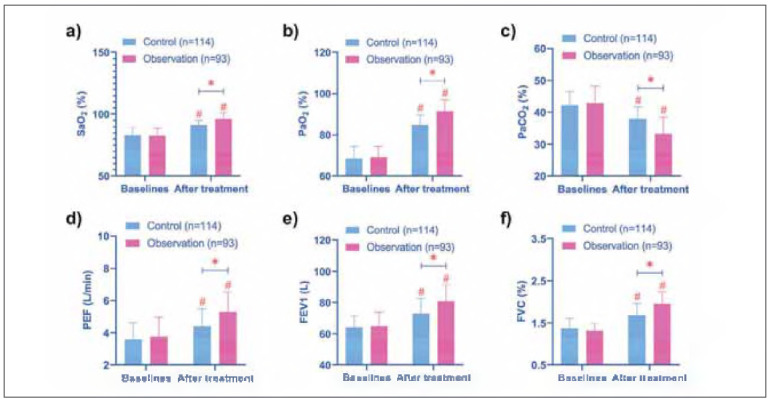
Comparison of pulmonary function. (a-f) show the comparison of SaO_2_, PaO_2_, PaCO_2_, PEF, FEV1 and FVC before and after treatment in the two groups, respectively. After treatment, SaO_2_, PaO_2_, PEF, FEV1 and FVC of the two groups increased, and the observation group was higher. However, PaCO_2_ decreased, and the observation group was even lower. # indicates P < 0.05 compared with baseline data, * indicates P < 0.05 compared between control and observation groups.

Post-treatment assessments revealed significant enhancements in pulmonary function in both groups. Key parameters such as PEF, FEV1, FVC, FEV1/FVC, SaO_2_, and PaO_2_ showed marked increases, while PaCO_2_ levels decreased (*P* < 0.05). Importantly, the observation group outperformed the control group in terms of FEV1, FVC, and PaO_2 _levels after treatment (*P* < 0.05) (Figure 1).

### Inflammatory mediators

**Figure 2:**
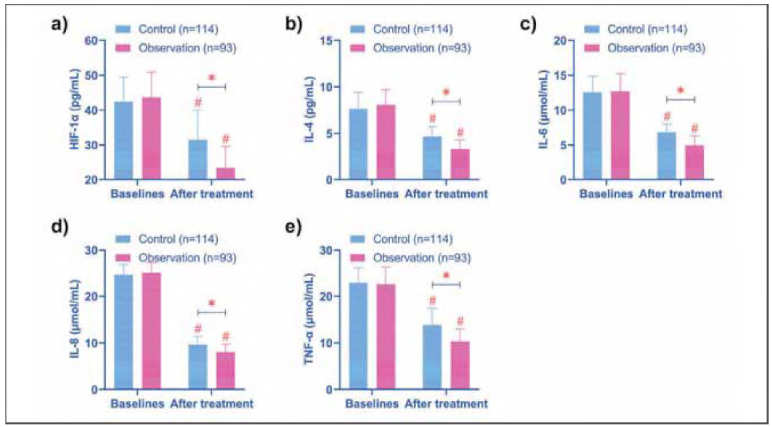
Comparison of inflammatory mediators. (a-e) show the comparison of HIF-1α, IL-4, IL-6, IL-8, and TNF-α before and after treatment in the two groups, respectively. After treatment, the levels of HIF-1α, IL-4, IL-6, IL-8, and TNF-α in the two groups were decreased, and the observation group was lower than the control group. # indicates P < 0.05 compared with baseline data, * indicates P < 0.05 compared between control and observation groups.

A significant reduction in inflammatory mediators was observed in both groups following treatment (*P* < 0.05). Notably, the observation group exhibited lower post-treatment levels of HIF-1α, IL-4, IL-6, IL-8, and TNF-α compared with the control group (*P* < 0.05) (Figure 2).

### Stress response

**Figure 3:**
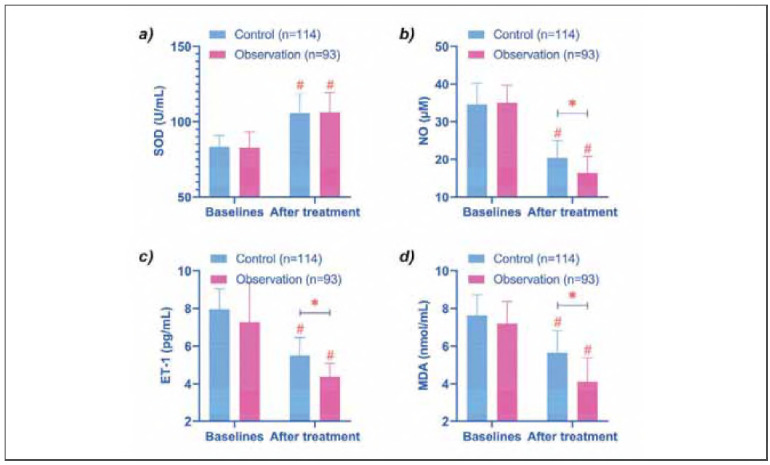
Comparison of stress response. (a-d) show the comparison of SOD, NO, ET-1, and MDA before and after treatment in the two groups, respectively. After treatment, SOD was increased in both groups. The levels of NO, ET-1 and MDA in the observation group were lower than those in the control group. # indicates P < 0.05 compared with baseline data, * indicates P < 0.05 compared between control and observation groups.

In terms of stress response indicators, SOD levels increased significantly from baseline in both groups after treatment (*P* < 0.05), with no notable difference between the groups (*P* > 0.05). Conversely, levels of NO, ET-1, and MDA decreased significantly in both groups post-treatment (*P* < 0.05). Moreover, the observation group achieved lower levels of these markers compared to the control group (*P* < 0.05) (Figure 3).

### Comparison of immunoglobulins

**Figure 4:**
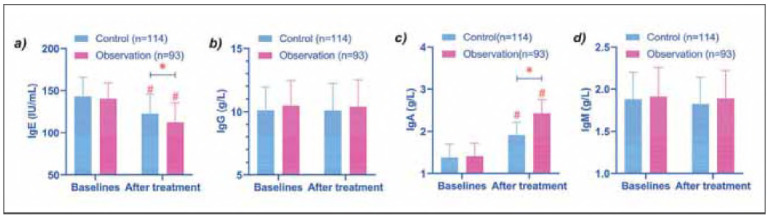
Comparison of immunoglobulins. (a-d) show the comparison of Ige, IgG, IgA, and IgM before and after treatment in the two groups, respectively. After treatment, the IgE of the two groups decreased, and the observation group was lower than the control group. However, IgA was elevated, which was higher in the observation group. # indicates P < 0.05 compared with baseline data, * indicates P < 0.05 compared between control and observation groups.

Finally, we measured immunoglobulin in both groups, and the results showed that no significant change in IgG and IgM levels after treatment in both groups. At the same time, there was no difference between the two groups (*P* > 0.05). However, IgE in the two groups after treatment was lower than that at baseline, and that in the observation group was lower than that in the control group (*P* < 0.05). However, the opposite was true for IgA (that is, it increased after treatment compared with baseline and was higher in the observation group than in the control group) (*P* < 0.05) (Figure 4).

## Discussion

Both SA and MP are well-established in clinical practice for BA treatment, with their therapeutic mechanisms thoroughly validated [13] [14]. MP primarily exerts its effects by inhibiting inflammatory factor secretion and reducing bronchial edema, while SA acts by dilating the bronchi and preventing the release of endogenous substances and inflammatory mediators [15] [16]. However, SA is not without limitations; its relatively slow onset of action and the risk of adverse effects due to excessive use, such as dyspepsia and tachycardia, remain significant concerns [17]. Although nebulization enhances the safety profile of SA, it further delays its therapeutic onset. For example, conventional salbutamol nebulization has poor therapeutic effect in some children [18], potentially attributable to this delayed action. The oxygen-driven SA approach effectively addresses this limitation. By delivering the drug directly to the airways and enhancing pulmonary drug deposition through oxygen carriage, this method achieves more rapid symptom relief. This aligns with the findings of Staedtke et al. [19], which lend further support to the results of this study. Moreover, the observation group exhibited significantly higher post-treatment FEV1 and PaO_2_ levels, highlighting the enhanced efficacy of this regimen in improving pulmonary ventilation and oxygenation in pediatric BA patients.

On the other hand, hypoxemia during acute exacerbations of BA has been shown to activate HIF-1 α, which subsequently promotes the secretion of pro-inflammatory factors such as IL-8 and IL-1β, exacerbating oxidative stress damage [20]. In this study, the observation group exhibited more pronounced alleviation in inflammatory mediators and stress responses, which can be attributed to several key factors: (1) Enhanced drug delivery efficiency: Oxygen-driven nebulization produces fine aerosol particles (1-5 μm) capable of penetrating deep into the small airways and alveolar regions, significantly increasing the local concentration of SA at distal inflammatory sites [21]. This mechanism not only directly inhibits airway smooth muscle spasms but also reduces mast cell degranulation and the release of inflammatory mediators, such as histamine and leukotrienes, thereby attenuating the activation of inflammatory signaling pathways [22]. In contrast, conventional nebulization generates larger particles, which predominantly deposit in the large airways, limiting their ability to regulate distal inflammation. (2) Synergistic effects of oxygen supply and anti-inflammatory/anti-stress mechanisms: The studies by Willson et al. demonstrated that inhibiting HIF-1α-mediated inflammatory cascades can effectively reduce neutrophil infiltration [23]. Additionally, reducing hypoxia-induced ROS generation alleviates lipid peroxidation and DNA damage [24]. Furthermore, alleviating respiratory muscle fatigue mitigates sympathetic nervous system excitation and HPA axis overactivation caused by respiratory distress [25], a mechanism particularly critical when combined with MP As highlighted by Xie et al. [26], MP suppresses inflammatory gene expression through genomic effects, while oxygen-driven nebulization may accelerate its anti-inflammatory onset via non-genomic pathways, such as improving the local microenvironment. (3) Improved treatment compliance and patient comfort: Clinical observations suggest that oxygen-driven nebulization, due to its shorter treatment duration (typically 30%-40% shorter than conventional nebulization) and simultaneous oxygen supply, alleviates wheezing symptoms and reduces crying and resistance in pediatric patients, thus indirectly lowering stress hormone release. In contrast, conventional nebulization, with its longer treatment time and lack of oxygen support, may exacerbate anxiety in children, leading to elevated stress markers such as adrenaline [27]. However, since this study did not specifically investigate treatment compliance, this perspective warrants further exploration. Regarding the long-term impact of oxygen-driven nebulization on inflammatory regulation (e.g., FeNO levels), existing studies present divergent views. For instance, Rius-Pérez et al. argue that its benefits are primarily evident during the acute phase, while chronic airway inflammation control still relies on the long-term, regular use of inhaled corticosteroids [28]. Given that the baseline data and post-treatment interval in this study spanned only 5 days, this perspective could not be validated.

Of note, this study also found that IgE was lower and IgA was higher in the observation group after treatment. IgE is the core mediator of allergic asthma, and its level is positively correlated with Th2-type inflammatory response [29]. In this study, the IgE decrease in the observation group was greater, suggesting that oxygen-driven administration may enhance the deposition efficiency of drugs in the deep airway, and more accurately inhibit mast cell degranulation and IgE-mediated cross-allergic reaction. Previous studies confirmed that airway mucosal sIgA levels are negatively correlated with airway hyperresponsiveness and serve as the first line of defense against pathogen invasion [30]. Oxygen-driven therapy may improve mucosal immunity through the following ways: ① High oxygen flow improves the hypoxia state of mucosal microenvironment and promotes the differentiation of IgA+ plasma cells [31]; ② The β2 receptor agonist effect of SA enhances the mucus secretion function of goblet cells and provides carrier support for sIgA [32].

Despite these findings, the retrospective design limits causal inference. Randomized controlled trials with longer follow-up periods are needed to validate the long-term safety and sustained efficacy of oxygen-driven nebulization. Furthermore, leveraging single-cell sequencing of bronchoalveolar lavage fluid could provide deeper insights into the regulatory mechanisms of oxygen-driven nebulization on key immune cell populations, such as airway macrophages and Th2 cells. However, this study did not distinguish between the allergen-specific and non-specific components of IgE, so it is difficult to accurately evaluate the effect of desensitization treatment. Second, short-term follow-up failed to track long-term changes in IgA subclasses, such as secretory versus serotype homeostasis, and these limitations need to be improved.

## Conclusion

The combination of oxygen-driven nebulized SA and MP demonstrates rapid symptom relief in pediatric BA, effectively curbing inflammatory and stress responses while enhancing pulmonary ventilation and immunoglobulins.

## Dodatak

### Availability of data and materials

The data that support the findings of this study are available from the corresponding author upon reasonable request.

### Funding

Not applicable.

### Conflict of interest statement

All the authors declare that they have no conflict of interest in this work.
